# Usefulness of dynamic volume scanning with 320-row CT in detecting recanalization of pulmonary arteriovenous fistula after coil embolization

**DOI:** 10.1186/2193-1801-2-169

**Published:** 2013-04-17

**Authors:** Ryoichi Tanaka, Kunihiro Yoshioka, Masayuki Takeda, Kenta Muranaka, Miyuki Sone, Michiko Suzuki, Shigeru Ehara

**Affiliations:** Department of Radiology, Iwate Medical University, 19-1Uchimaru, Morioka, Iwate 020-8505 Japan; Department of Diagnostic Radiology, National Cancer Center, 5-1-1 Tsukiji Chuo-ku, Tokyo, 104-0045 Japan

**Keywords:** Pulmonary arteriovenous fistula, Dynamic scan, MDCT, Coil embolization, Recanalization

## Abstract

Pulmonary arteriovenous fistula is a congenital and rarely acquired anomalous direct communications between pulmonary arteries and veins. Transcatheter embolization using metallic coil or detachable balloon is one of the common treatment procedure. However, recanalization after the embolization is one of the concern and its differentiation from the retrograde filling via pulmonary vein is difficult except using invasive angiography. We report a case with recanalized pulmonary arteriovenous fistula non-invasively detected by dynamic CT angiography with 320-rows multi detector CT.

A 45-year-old women who had underwent coil embolization for pulmonary arteriovenous fistula was examined with dynamic CT angiography and antegrade contrast enhancement of the fistula was noted. The recanalization through the embolized artery was confirmed by digital subtraction angiography, and the second coil embolization was performed. The follow-up dynamic CT angiography at three months after the second procedure found the retrograde enhancement of aneurysmal sac and no antegrade shunt. The dynamic CT angiography was useful for the detect the recanalization of pulmonary arteriovenous fistula.

Delayed pulmonary artery recanalization was reported to be observed in 5- 10% of cases as a complication after the successful occlusion of segmental pulmonary artery. Lack of change in aneurysmal diameter of pulmonary arteriovenous fistula demonstrated by CT was reported as the result of persistent aneurysmal perfusion or aneurysmal thrombosis. However, the retrograde filling of aneurysmal sac via pulmonary vein or remnant collateral pathway to the pulmonary arteriovenous fistula were also considered. Therefore, before the invasive procedure, we performed dynamic CT angiography to detect the flow direction and pathway to the pulmonary arteriovenous fistula. Using dynamic CT angiography, we could obtain hemodynamic information through the aneurysmal sac of pulmonary arteriovenous fistula and decide to proceed to the invasive embolotherapy. Prospective perfusion CT scan could be an alternative to invasive angiography in the initial follow-up after the embolotherapy or in the cases with the recanalization of pulmonary arteriovenous fistula.

**Electronic supplementary material:**

The online version of this article (doi:10.1186/2193-1801-2-169) contains supplementary material, which is available to authorized users.

## Background

Pulmonary arteriovenous fistula (PAVF) is a congenital and rarely acquired anomalous direct communications between pulmonary arteries and veins. Transcatheter embolization using metallic coil or detachable balloon is one of the common treatment procedure. The follow-up of PAVF after the embolization is usually done by chest plain X-ray or CT, however, the direct visualization of rencanalization through PAVF by contrast enhanced CT or MRI is difficult, because of the severe metallic artifact by embolization coils or retrograde filling of aneurysmal sac. Catheter angiography is the most reliable diagnostic procedure for the detection of recanalization, but invasive. This report describes the non-invasive detection of recanalization of PAVF by CT angiography with dynamic volume scanning (dynamic CTA) using 320-rows MDCT.

## Case description

**Figure 1:**
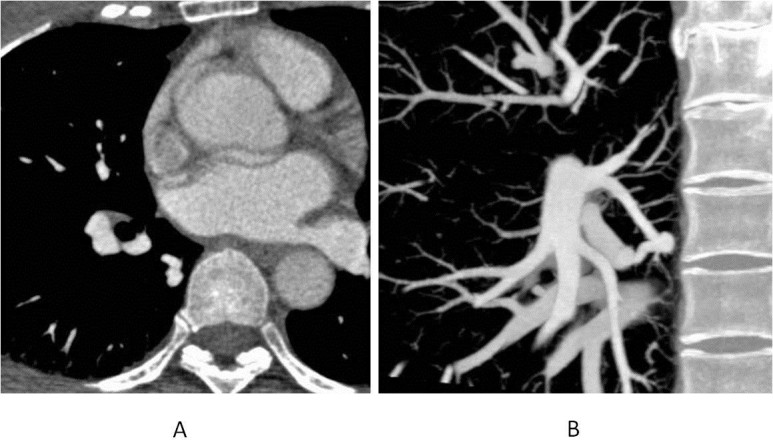
**Pulmonary arteriovenous fistula (PAVF) found on the conventional contrast-enahnanced CT. A**: On axial image of contrast-enhanced CT, aneurysmal sac of PAVF was noted at S6 of right lower lobe. **B**: Para sagital oblique view of partial maximum intensity projection (MIP) shows the anomalous continuation of pulmonary artery and vein with aneurysmal sac.

**Figure 2:**
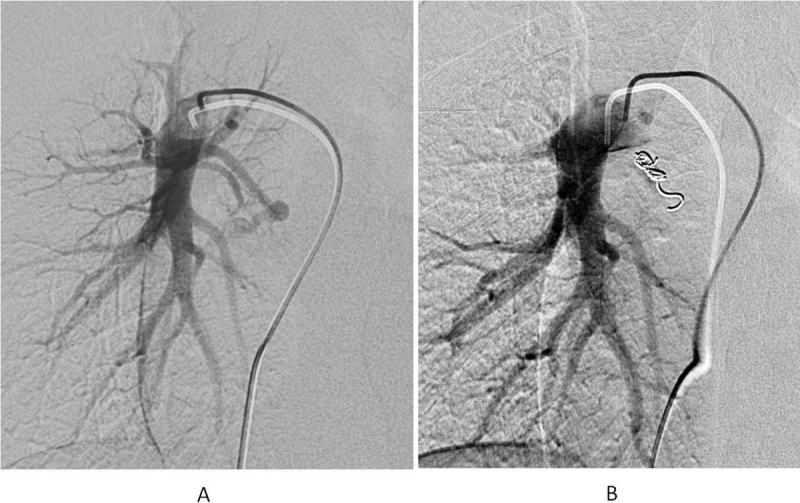
**Transcatheter coil embolization. A**: PAVF at right lower lobe (S6) was noted. **B**: After the embolization, no residual shunt was noted.

A 45-year-old women with chest X-ray abnormality referred our department for further evaluation and treatment. Contrast enhanced CT revealed the solitary PAVF at lower lobe of right lung (Figure 1). SpO_2_ under the room air was 98%. Transcatheter coil embolization for PAVF with a 0.035-inch embolization coil (3-5 mm) and micro coils (3 mm × 4, 4 mm × 1) was performed and complete occlusion of PAVF was achieved (Figure 2). On the follow-up contrast enhanced CT after 6-month from the first procedure, the aneurysmal sac of PAVF was still enhanced and its diameter (6.3 mm) was not remarkably changed. For the differentiation between recanalization and retrograde filling of the sac, we conducted the dynamic volume scanning by 320-rows MDCT (Aquilion one, Toshiba Medical Systems, Japan), because of the less invasiveness in comparison with conventional angiography. The dynamic CTA was achieved with the injection of 40-mL contrast media (Iopamidol 370 mgI/mL) followed by 35-mL saline flush at the rate of 4 mL/sec from right antecubital vein. To reduce the radiation dose, we used 100-kV tube voltage and the scan length was limited to 100 mm (Khan et al. 2011) (200-rows mode with 0.5-mm slice thickness) for the inclusion of the PAVF and parent vessels. Other scan parameters were shown on Table 1. The dose length product (DLP) of this study was 809 mGy.cm and the effective radiation dose was 11 mSv derived from the sum of DLP multiplied by CT conversion coefficient (k = 0.014 mSv/mGy × cm) (Raff et al. 2009).

**Table 1:** **Parameters of dynamic scanning**

Parameter	Value
Scan mode	Dynamic volume scan
Slice thickness	0.5 mm
Scan length	100 mm (200 rows)
Gantry rotation speed	0.35 sec/rotation
Scan interval	1 sec
The number of scan	8 times
Tube voltage	100 kV
Tube current	350 mA

**Figure 3:**
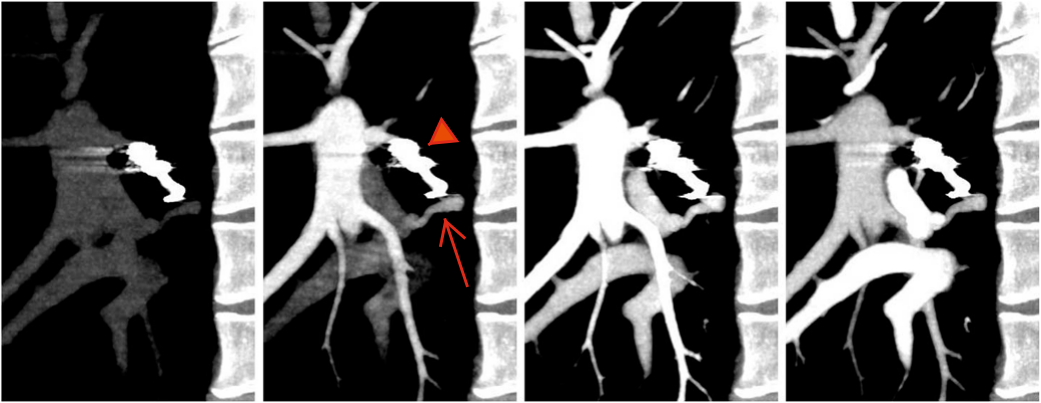
**Dynamic CTA after the first intervention using dynamic volume scan with contrast enhancement.** Early contrast filling and wash-out of aneurysmal sac of PAVF was noted. Enhancement of aneursymal sac was almost same as that of pulmonary artery (arrow) and suggested recanalization through the embolization coil (arrow head).

**Figure 4:**
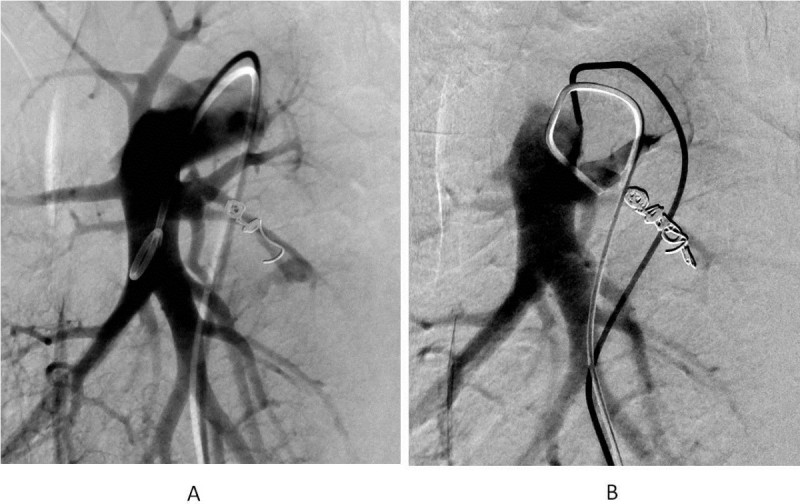
**Secondary transcatheter coil embolization. A**: Recanalization through previously inserted coils was noted. **B**: After the additional coil embolization, no residual shunt was noted.

**Figure 5:**
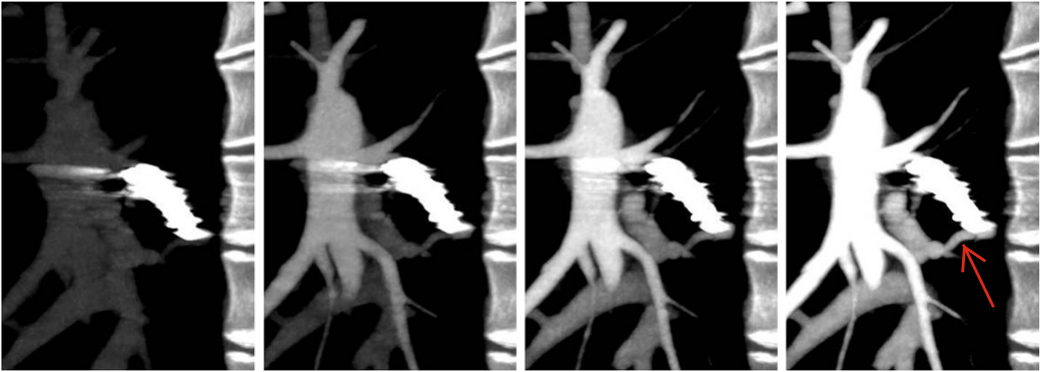
**Dynamic CTA after the secondary intervention.** No early contrast filling in the aneurysmal sac of PAVF was noted. The aneurysmal sac of PAVF was enhanced at the same phase of the enhancement of pulmonary vein (arrow).

The aneurysmal sac of PAVF was enhanced at the same phase of pulmonary artery enhancement observed, and this findings meant the antegrade recanalized blood flow through the coil to the fistula (Figure 3, Additional file 1). Based on this findings, secondary intervention was achieved for embolizing the remnant lumen with embolization coils (a 4 mm interlocking detachable coil and a 3-4 mm micro embolization coil). The recanalization through the embolized artery was found by digital subtraction angiography (DSA), and secondary embolization with detachable micro coils were achieved. No remnant shunt was observed after the coil embolization (Figure 4). The follow-up dynamic CTA at three months after the secondary procedure showed a delayed enhancement of the aneurysmal sac after the phase of pulmonary vein enhancement (Figure 5, Additional file 2) with regression in aneurysmal diameter (5.9 mm).

Additional file 1: Movie of dynamic CTA after the first intervention using dynamic volume scan with contrast enhancement. (MP4 404 KB)Additional file 1: Movie of dynamic CTA after the second intervention. (MP4 292 KB)

## Discussion

PAVFs are congenital or acquired abnormal direct connections between pulmonary arteries and veins. Most of the patients are asymptomatic, however, PAVF is a cause of dyspnea with arterial hypoxia, cyanosis, hemoptysis, heart failure and various central nervous system complications such as transient ischemic attacks, seizures, stroke, and brain abscess due to paradoxical embolization (Pick et al. 1999). Transcatheter embolization of PAVF is now a well-accepted procedure (Remy-Jardin et al. 1991). Recently, Amplatzer vascular plug are used for the embolization of large PAVF (Rossi et al. 2006), however, metallic coils and occlusion balloons have a major role for interventional occlusions of small or medium-sized PAVFs (Liau et al. 1997; Rankin et al. 1983).

Delayed pulmonary artery recanalization was reported to be observed in 5- 10% of cases as a complication after the successful occlusion of segmental pulmonary artery (Remy-Jardin et al. 1991; White and Pollak 1994). Lack of change in aneurysmal diameter of PAVF demonstrated by CT was reported as the result of persistent aneurysmal perfusion or aneurysmal thrombosis (Remy et al. 1992). As a result, the reduction of aneurysmal diameter was observed after the second procedure, however, the change of the diameter was only 0.4 mm and the change was less than the size of one pixel of the CT (0.5 mm). A little change in aneurysmal diameter often makes it difficult to decide advancing to the additional invasive procedure. Also, when the enhancement in aneurysmal sac was observed, the retrograde filling of aneurysmal sac via pulmonary vein, remnant supply from small pulmonary artery, or collateral pathway from bronchial artery to the PAVF should be differentiated, because the recanalization and remnant supply require the additional intervention but the others are not. Conventional one or two-phase contrast enhanced CT cannot distinguish the direction of blood flow. Therefore, dynamic CTA is necessary to detect the flow direction and pathway to the PAVF before the invasive procedure, when the change of diameter of aneurysmal sac was little and the enhancement of aneurysmal sac was observed.

Using 320-rows CT, perfusion scanning by wide-coverage detector without the movement of patient’s table can be achieved. For PAVF, simultaneous observation of parent pulmonary artery and vein is achievable with this wide-coverage detector. The z-axis coverage of 64 rows CT is only 3 cm, and makes it difficult to keep both aneurysmal sac and parent vessels in the same field of view during the dynamic scanning when the patient’s breathholding is unstable.

Because of the metallic artifact from the metallic embolization coils, direct imaging of the recanalization cannot be achieved even in the perfusion scanning by 320-rows CT. However, the relation of contrast-enhancement between the aneurysmal sac and pulmonary circulation could reveal the effectiveness of embolotherapy. In this case, the enhancement of aneurysmal sac in the pulmonary arterial phase was observed. This finding suggested the renanalization or remnant supply from adjacent small pulmonary artery. Angiography obtained during the second intervention proved antegrade blood flow through the previously implanted coils as the dynamic CTA revealed. After the second intervention, the aneurysmal sac of PAVF was not enhanced in the pulmonary arterial phase but enhanced in the pulmonary venous phase, and this findings supported that the effective embolotherapy had been achieved.

Radiation dose of CT is a subject to discuss, especially in the perfusion study. We used the intermittent exposure in perfusion scan mode to minimizing the radiation dose. The estimated radiation dose in this study was 11 mSv and did not exceed the reported maternal dose (7 - 28 mSv) of DSA in pregnancy with pulmonary thromboembolism as a diagnostic test (Leung et al. 2012). Prospective perfusion CT scan could be an alternative to invasive angiography in the initial follow-up after the embolotherapy or in the cases with the recanalization of PAVF suspected.

## Conclusion

In conclusion, prospective perfusion scan by 320-rows CT was useful in the detection of recanalization and follow-up of PAVF.

### Ethical committee approval and consent

Our institutional ethical committee approved this study and written informed consent was obtained from the patient for publication of this report and any accompanying images.

## Authors’ original submitted files for images

Below are the links to the authors’ original submitted files for images. Authors’ original file for figure 1 Authors’ original file for figure 2 Authors’ original file for figure 3 Authors’ original file for figure 4 Authors’ original file for figure 5

## Abbreviations

CT: Computed tomography
MRI: Magnetic resonance imaging
CTA: Computed tomographic angiography
MDCT: Multidetector CT
DSA: Digital subtraction angiography
PAVF: Pulmonary arterio-venous fistula
DLP: Dose length product.
