# Supplementary figures and images for: Usefulness of dynamic volume scanning with 320-row CT in detecting recanalization of pulmonary arteriovenous fistula after coil embolization
Source: Springerplus. 2013 Apr 17;2(1):169. doi: 10.1186/2193-1801-2-169 (PMC3664737; doi:10.1186/2193-1801-2-169)

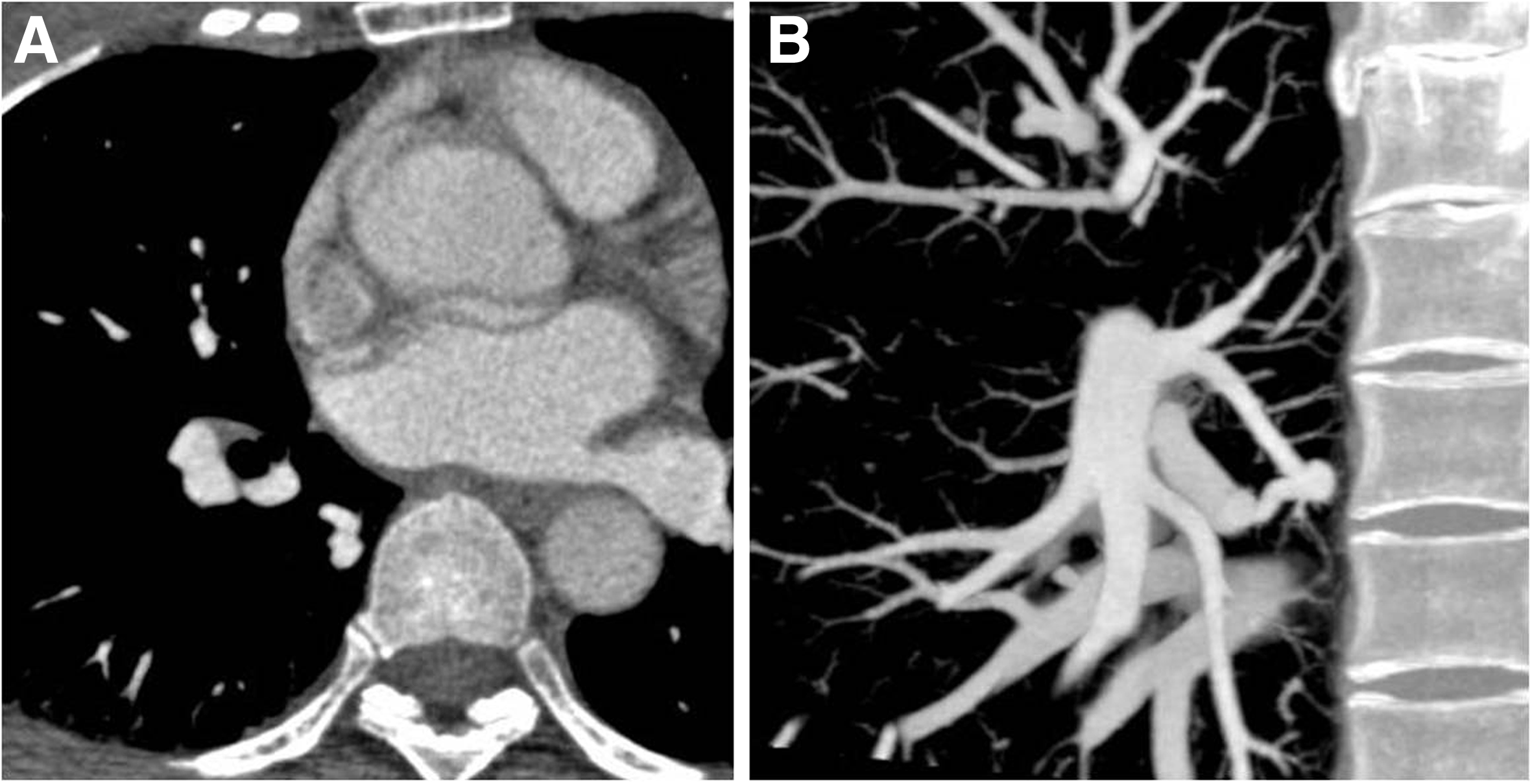

Supplement: Supplementary file 3 — Authors’ original file for figure 1 [file 40064_2013_279_MOESM3_ESM.tiff]

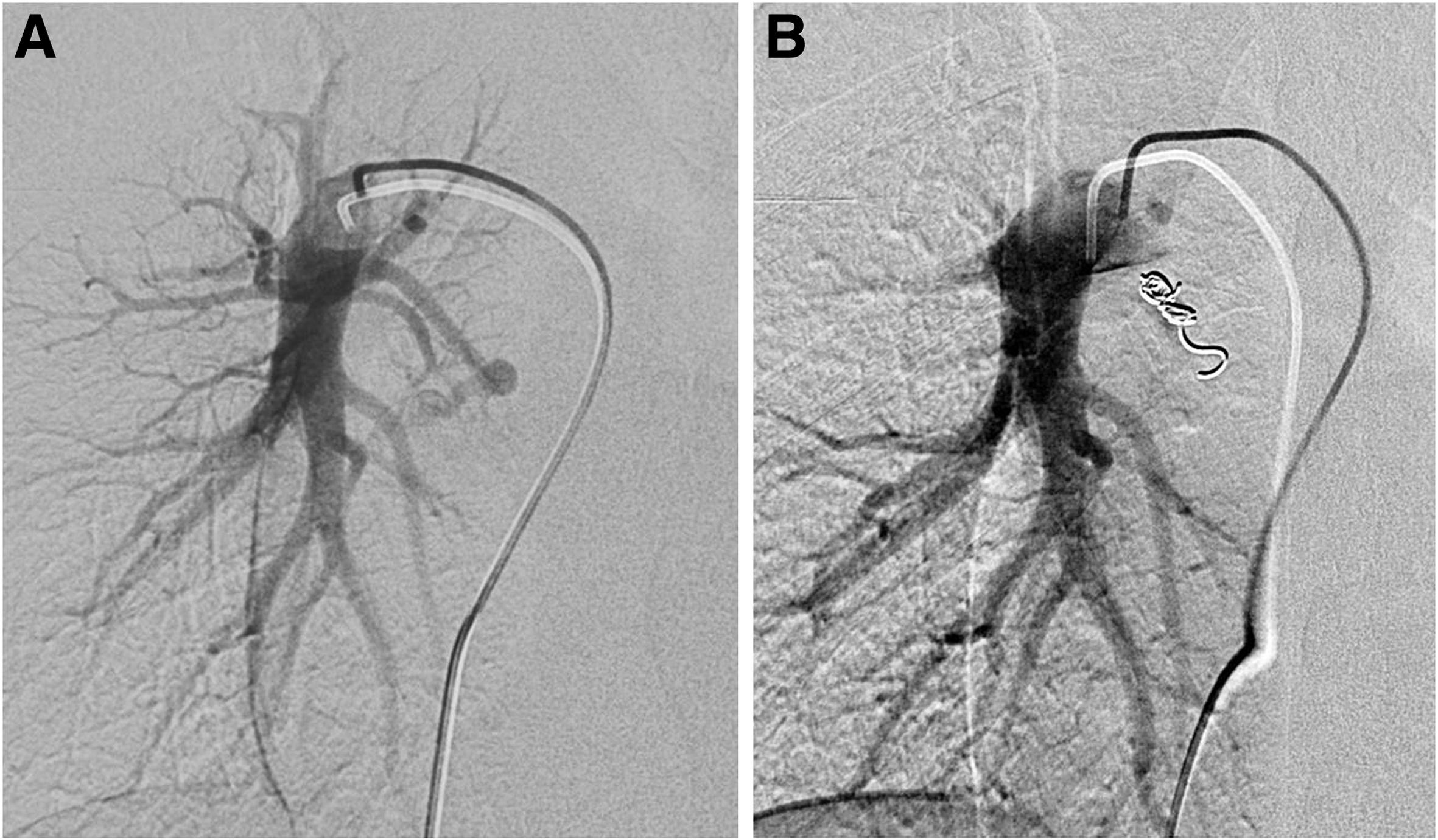

Supplement: Supplementary file 4 — Authors’ original file for figure 2 [file 40064_2013_279_MOESM4_ESM.tiff]

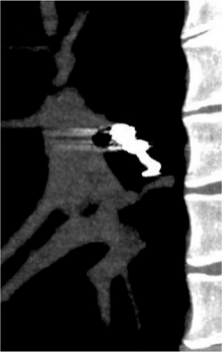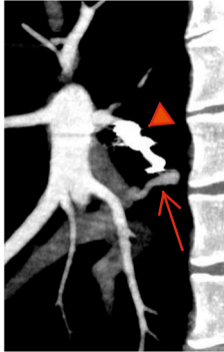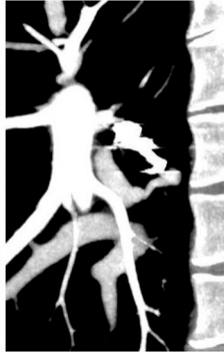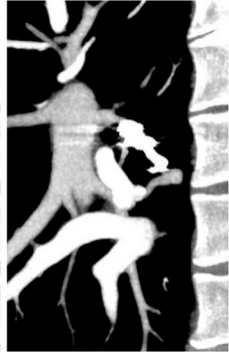

Supplement: Supplementary file 5 — Authors’ original file for figure 3 [file 40064_2013_279_MOESM5_ESM.pdf]

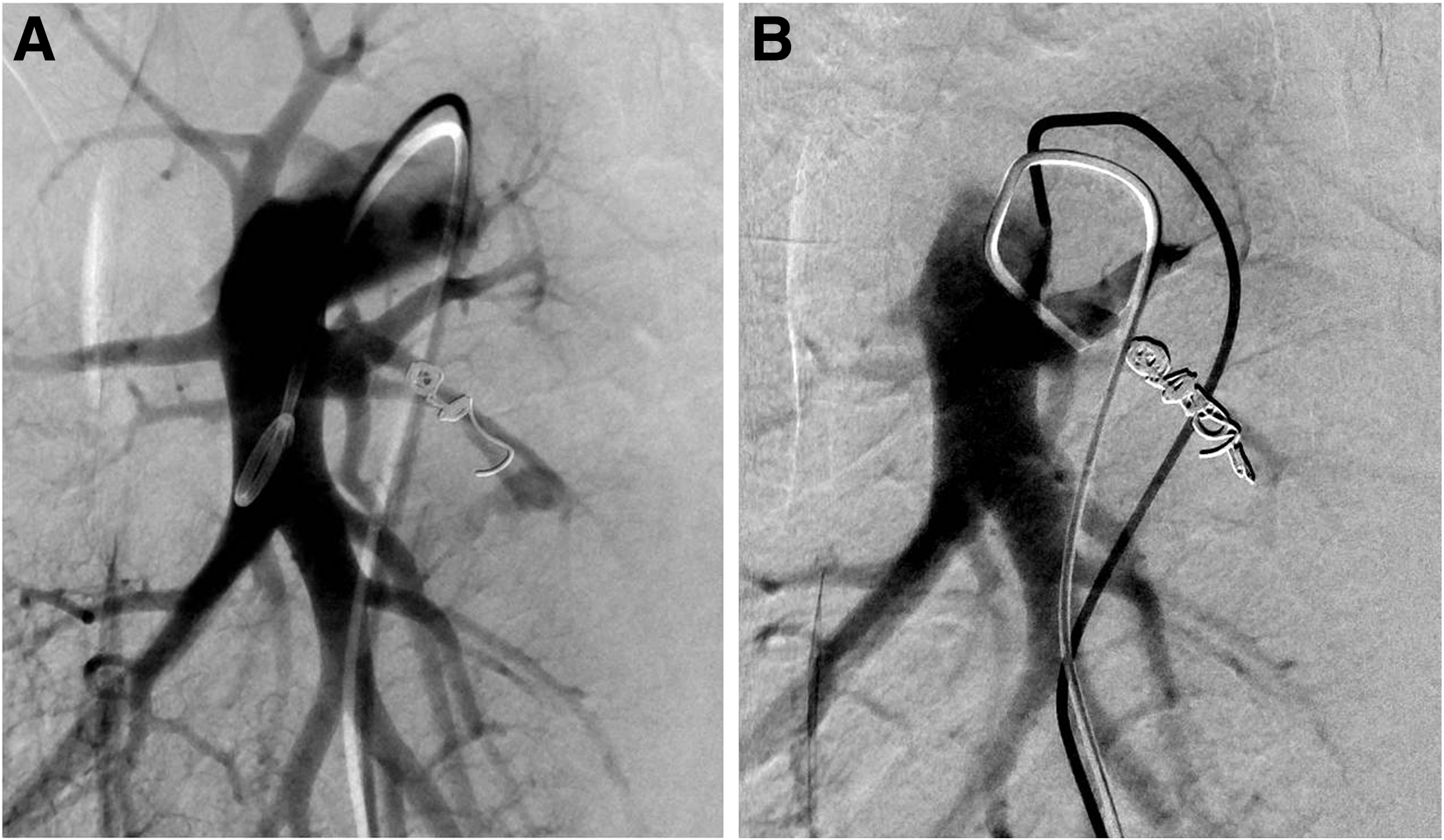

Supplement: Supplementary file 6 — Authors’ original file for figure 4 [file 40064_2013_279_MOESM6_ESM.tiff]

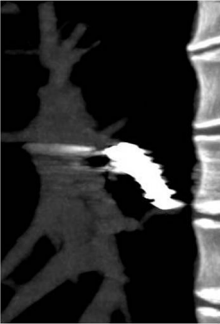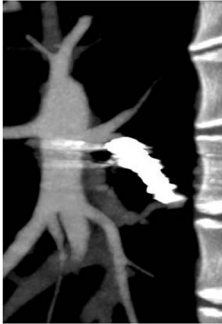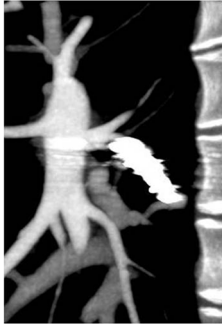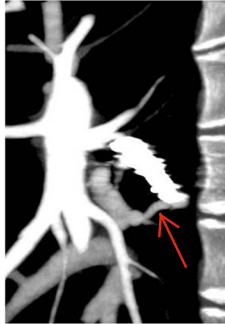

Supplement: Supplementary file 7 — Authors’ original file for figure 5 [file 40064_2013_279_MOESM7_ESM.pdf]
